# Modal Analysis–Based Detection of Barely Visible Impact Damage in Carbon/Epoxy Overwraps of Type-IV Polymer-Lined Pressure Vessels

**DOI:** 10.3390/polym17223068

**Published:** 2025-11-19

**Authors:** Mirosław Bocian, Mikołaj Kazimierczak, Barbara Kmiecik, Marek Kryspin, Maciej Panek

**Affiliations:** 1 Department of Mechanics, Materials and Biomedical Engineering, Faculty of Mechanical Engineering, Wroclaw University of Science and Technology, Smoluchowskiego 25, 50-372 Wroclaw, Poland; miroslaw.bocian@pwr.edu.pl (M.B.); mikolaj.kazimierczak@pwr.edu.pl (M.K.); barbara.kmiecik@pwr.edu.pl (B.K.); 2 Faculty of Pure and Applied Mathematics, Wroclaw University of Science and Technology, Wybrzeże Wyspiańskiego 27, 50-370 Wroclaw, Poland; marek.kryspin@pwr.edu.pl

**Keywords:** modal analysis, composite materials, damage detection, high-pressure vessels, vibration analysis, finite element method

## Abstract

A vibration-based protocol is presented for identifying barely visible impact damage (BVID) in type-IV composite-overwrapped pressure vessels (COPVs). A 1 kJ hemispherical-tip strike was applied to a fully pressurized vessel, which was subsequently depressurized and characterized by free–free experimental modal analysis over a 168-point grid. The frequency response functions (FRFs) at the impact meridian exhibited distinct peaks near 3.70, 4.34, and 4.90 kHz with larger amplitudes and lower coherence than at the diametrically opposite meridian, indicating local circumferential stiffness loss. A detailed finite element model of the liner, bosses, and carbon/epoxy overwrap was updated by idealizing a cylindrical sub-volume with a 90% reduction in orthotropic stiffness. The pristine and “damaged” numerical modal sets agreed closely (mean frequency error < 2%), and for most of the first 60 modes, the diagonal Modal Assurance Criterion (MAC) remained ≥ 0.90. However, in several nearly degenerate circumferential mode pairs, the diagonal MAC dropped to 0.49–0.88 because the local asymmetry rotated the eigenvectors within a common subspace, showing that classical MAC alone cannot expose such early-stage defects. Radial displacement scan-lines provided the missing spatial resolution. Modes whose antinodal regions intersect the dent showed pronounced local amplitude bulges and slight angular shifts in the peak toward the impact site, whereas modes with a nodal line across the damage were virtually unchanged. The combined use of FRF asymmetry, MAC screening, and scan-line deformation profiling localized the impact to the correct circumferential sector with centimeter-scale resolution along the scan ring, yielding predictive signatures for rapid, non-pressurized in situ assessment of impacted COPVs after depressurization.

## 1. Introduction

Composite-overwrapped pressure vessels (COPVs) are widely used for high-pressure gas storage. In type-IV designs, a polymer liner is overwrapped with carbon-fiber/epoxy, which provides high mass efficiency [1]. Accidental impacts can introduce barely visible damage, such as matrix cracking and delamination [2], and reduce the residual burst pressure of composite vessels [3]. These risks motivate non-destructive, vibration-based identification of impact damage [4].

COPVs are exposed to accidental low-velocity impacts during handling and service, for example, drops, tool strikes, or forklift contact [1]. These events can generate matrix cracking, fiber breaks, and interlaminar delamination that often escape visual inspection [2,5]. Such barely visible damage reduces the residual burst strength of the composite cylinders, which motivates reliable non-destructive evaluation [3,5].

Experimental modal analysis (EMA) provides a purely vibration-based route to damage assessment. Variations in natural frequencies, damping, and mode shape curvature correlate with local stiffness loss in laminated structures [6,7,8,9]. Recent methods leverage mode shape derivatives: for example, Kindova-Petrova [10] introduced the Mode Shape Slope Difference (MSSD) index, which pinpoints damage locations in beams by comparing forward- and backward-difference slopes of a single mode shape. Manoach et al. [11] review modal-based damage detection methods (including modal displacement, slope, curvature, and strain-energy approaches) and compare their effectiveness via finite element and experimental studies. Compared with hydrostatic proof testing, EMA is quicker, less expensive, and can be performed without repressurizing the vessel [5]. Beyond classical frequency and mode shape indicators, FRF-based damage metrics derived from swept-sine tests with embedded piezoelectric transducers have been shown to quantify damage growth, extending vibration-based SHM from detection to size estimation [12]. Note that operational modal analysis (OMA) can also detect subtle defects [13], but the present work focuses on EMA with controlled excitation. Nevertheless, systematic studies devoted to high-pressure composite vessels remain relatively few compared to plates and beams [14].

Recent reviews highlight that the resolution of vibration-based structural health monitoring strategies increases when experimental data are interpreted through validated numerical models [5,15]. High-fidelity finite element (FE) representations of COPVs covering wound shells and boss regions have been developed and validated against tests, enabling controlled studies of stiffness reductions [16,17,18]. Three-dimensional continuum-damage models now allow end-to-end simulation of linerless vessels [19]. Recent work also links modeling with data-driven methods and structural monitoring: FE plus XGBoost optimization has been demonstrated on pressure vessels [20], and embedded optical sensing has been integrated during the manufacture of COPVs [21], while a comprehensive review survey advances in modeling and design for hydrogen storage vessels [22]. Related data-centric SHM advances have been reported in large civil structures, including data augmentation and adaptive optimization [23,24]. For complex shell-type structures, robust mode correlation is critical; combined mode, strain energy-based indices that employ MAC-guided matching and interpolation have improved localization accuracy under measurement noise and modal shifts [25].

To quantify stiffness changes, the Modal Assurance Criterion (MAC) provides a well-established measure of mode-shape similarity [26,27]. In the present work, MAC is applied solely to the numerical mode sets of pristine and damaged FE models.

Complementary investigations demonstrate the breadth of vibration-based diagnostics. Guided-wave studies confirm the technique’s capability for global vessel monitoring and inform the transducer network design on COPVs [28].

Vibration tests on fluid-filled filament-wound cylinders show that dynamic methods remain viable under service conditions and can be implemented with residual-based OMA testing [29]. A full-field laser-scanning vibrometer localized internal flaws in circular cylinders with submillimeter precision by exploiting spatial derivatives of operating deflection shapes [30].

Against this background, this study investigates whether a combined EMA–FE framework can detect and locate barely visible impact damage in type-IV COPVs using modal shapes asymmetries and scan-line deformation patterns, separate global mode-ordering effects from local stiffness reductions via MAC screening on numerical mode sets, and provide practical expectation sets for modal testing of vessels of unknown condition after accidental drops without depressurization. The approach integrates free–free EMA with a validated FE model parameterized by local stiffness knock-downs, establishing a basis for interpreting measured responses under plausible damage scenarios.

## 2. Materials and Methods

The research strategy integrates controlled-impact testing with physics-based finite-element (FE) simulation to quantify how barely visible impact damage alters the dynamic behavior of a type-IV composite-overwrapped pressure vessel (COPV). A 1 kJ drop tower strike was applied to a fully pressurized vessel, which afterwards was characterized by free-free experimental modal analysis (EMA) on a 168-point excitation grid arranged in seven evenly spaced circumferential rings. The response was measured by a single accelerometer fixed at a reference location, while the modal hammer was roved across the grid points.

A commercial off-the-shelf type-IV COPV from Hexagon Composites (Lincoln, NE, USA) served as the test article, specified at 36 L nominal water capacity and 70 MPa working pressure. The vessel geometry was later reproduced in an FE model that resolves the aluminum bosses, HDPE liner, and a homogenized carbon/epoxy overwrap. The impact-affected zone was idealized as a cylindrical sub-volume with a 90% reduction in orthotropic stiffness. This severe, local knock-down was adopted as an upper-bound sensitivity case to accentuate damage-driven FRF and shape-based signatures relative to experimental variability and modeling error, and to bracket detectability while retaining global mode ordering. This modeling choice provides a clear contrast for assessing diagnostic indicators across plausible BVID scenarios rather than representing a single physical state.

Two complementary diagnostics were used: the diagonal Modal Assurance Criterion (MAC) was computed between pristine and damaged vessel numerical eigenvectors to verify mode pairing and isolate the effect of local stiffness loss on mode topology and a set of virtual scan-lines; two longitudinal meridians and seven circumferential rings, one passing through the indentation center, was defined on the mesh and additionally marked on the vessel. Sampling radial displacement along these paths yields one-dimensional deformation profiles. The comparison between pristine and damaged profiles from FE is intended to reveal local amplitude changes, phase shifts, and curvature variations that locate the affected sector and inform sensor placement for subsequent monitoring.

### 2.1. Experimental Research

The experiment was designed to evaluate the vessel’s structural integrity under impact loading and to identify dynamic responses using experimental modal analysis (EMA).

In the first stage, the vessel was subjected to controlled impact loading to simulate random mechanical impacts that can occur during transport, handling, or operational incidents. The impact was applied to the central region of the cylindrical section of the vessel using a drop tower setup equipped with a semi-spherical steel impactor.

**Figure 1:**
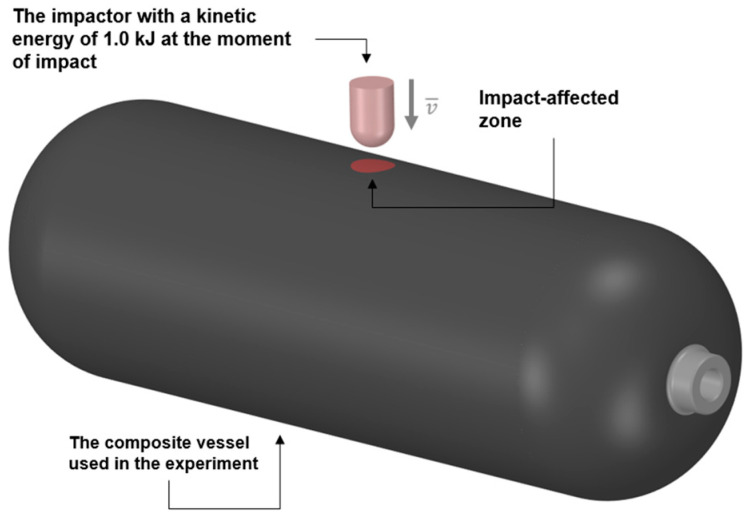
Schematic of the impact test.

**Figure 2:**
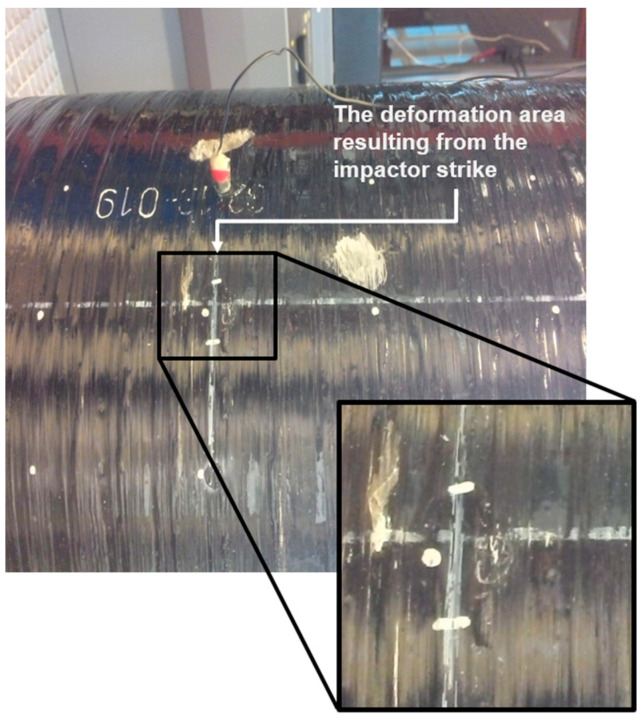
Photograph of the COPV after impact. The enlarged inset marks the barely visible indentation produced by the 1 kJ hemispherical impactor.

The drop tower’s height and the mass of the impactor were adjusted to deliver a total impact energy of 1 kJ, with 0.6 kJ being absorbed by the vessel. During the test, the vessel was pressurized to its working pressure of 70 MPa using compressed air. The impact location was carefully chosen to represent a critical zone for stress propagation in the vessel’s structure (Figure 1). Pre- and post-impact inspections were carried out visually to detect any visible signs of damage, such as cracking or delamination (Figure 2).

**Figure 3:**
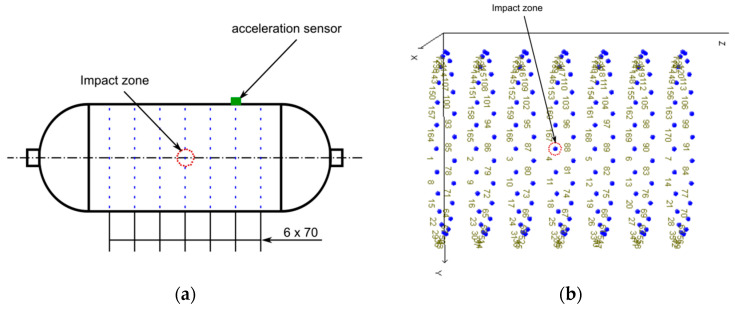
Experimental measurement grid. (**a**) Overall geometry showing the impact zone and the reference accelerometer mounted 90° circumferentially from the impact meridian. (**b**) Distribution of the 168 excitation points arranged in seven evenly spaced circumferential rings (blue).

In the second stage, experimental modal analysis (EMA) was performed to characterize the dynamic response of the impacted vessel and extract indicators sensitive to local stiffness reductions, after the vessel had been depressurized to ambient level to avoid pressure-induced shifts in the measured natural frequencies. The vessel was suspended vertically using soft nylon ropes to approximate free–free boundary conditions. Seven evenly spaced circumferential rings were marked on the cylindrical section; each ring comprised 24 points at 15° azimuthal spacing, providing uniform surface coverage (Figure 3). The measurements were acquired with a roving instrumented hammer and a single response accelerometer fixed at a reference location.

The vessel was excited with a lightweight modal hammer (PCB Piezotronics 086C03, Depew, NY, USA; head Ø 15.7 mm, hammer mass 0.16 kg, with hard tip), and the structural response was recorded by a miniature accelerometer (Isotron 35C-10, Endevco, Halifax, NC, USA); nominal sensitivity 10 mV/g, sensor mass 1.7 g) bonded at Point 134 on Ring 6, positioned 90° from the impact meridian to minimize mass loading. This sensor served as the fixed response channel for the campaign. The hammer force vector and the accelerometer measurement axis were oriented normal to the vessel wall to measure the out-of-plane component. Signals were acquired with an eight-channel Dewesoft SIRIUS analyzer with Dewesoft X3 software (Dewesoft, Trbovlje, Slovenia). Each of the 168 excitation points was struck three times to obtain an averaged FRF at each measurement point. From the averaged FRFs, natural frequencies, damping ratios, and mode shapes were identified.

### 2.2. Methodology of Numerical Simulations

**Figure 4:**
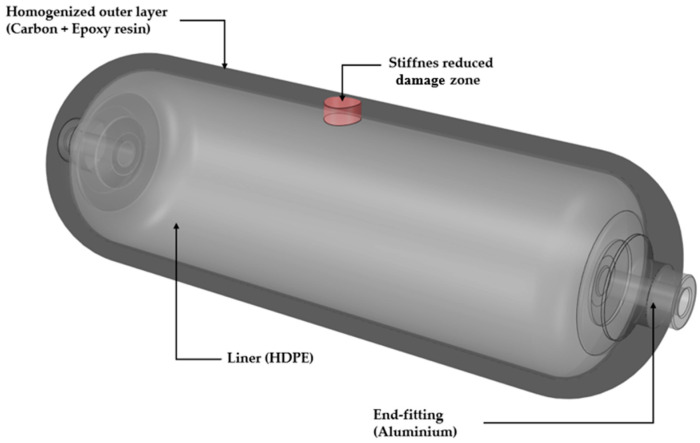
Material domains represented in the finite-element model. The type-IV architecture comprises an HDPE liner, aluminum end-fittings, and a homogenized carbon/epoxy over-wrap. Impact volume is idealized as a cylindrical sub-volume with a 90% stiffness knock-down.

A three-dimensional finite-element model of the Type-IV vessel was created in ANSYS Mechanical. The assembly comprises aluminum end-fittings, an HDPE liner (The physical properties of these materials are listed in Table 1), and a carbon/epoxy overwrap (Figure 4).

Because the present study focuses on global stiffness rather than local fiber paths, the overwrap was homogenized to an orthotropic lamina whose elastic constants reproduce the longitudinal/transverse anisotropy. The FE damage region was placed at the experimentally observed impact meridian, mid-span. Its through-thickness extent covered the entire carbon/epoxy overwrap as an envelope case, consistent with effective stiffness surrogates for distributed interlaminar damage and with the high bending sensitivity of composite cylindrical shells to interlaminar degradation [31,32]. In planform, the damage-zone diameter was chosen conservatively as ~10× the visible dent to probe detectability; subsurface delamination in CFRP commonly extends well beyond the surface indentation and typically scales with indentation depth or impact energy [33,34,35]. The use of a uniform stiffness reduction patch as a practical surrogate for localized impact damage in vibration/FE studies is established, with local stiffness change zones inserted parametrically to emulate defects for modal screening and inverse identification [36,37].

The carbon/epoxy overwrap was modeled as a cylindrically orthotropic, hoop-dominated laminate. The circumferential modulus was set to 140 GPa, i.e., within the 135–150 GPa range reported for hoop-wound carbon/epoxy rings at $V_{f}\approx 0.55–0.6$ and consistent with rule-of-mixtures estimates for 230–240 GPa fibers at this fiber volume fraction [38,39]. Initial elastic constants were taken from published characterizations of hoop-wound CFRP cylinders and from IM7/8552 or T700/epoxy datasheets and were subsequently modified in a controlled way to increase modal sensitivity to circumferential stiffness loss. Accordingly, material constants were adopted from studies on filament-wound pressure vessels, where equivalent orthotropic properties are identified from mechanical or internal pressure tests [40,41], and from public UD prepreg data providing transverse and shear moduli for IM7/8552 and T700/epoxy systems [39,42].

The axial and radial moduli were then intentionally reduced to 8 GPa to represent matrix-controlled directions and to isolate the effect of the circumferential–non-circumferential stiffness contrast on the modal response, following identification-oriented FE studies that downscale non-dominant moduli to enhance sensitivity to local hoop softening [40], while preserving a stiffness hierarchy consistent with filament-wound composite pressure vessels designed for internal-pressure loading [43].To keep the stiffness matrix positive definite for $E_{\theta }\gg E_{r}$, Poisson’s ratios involving the radial direction were capped at 0.02, which is a standard choice for highly anisotropic wound shells, whereas the in-surface coupling $\nu_{\theta z}$ was retained at 0.34, consistent with reported values for wound pipes and cylinders [38,41]. All shear moduli were set to 5 GPa, which lies within the 4–6 GPa band for IM7/8552 and T700/epoxy UD systems and is acceptable when the exact layup is unknown [39,42]. The density in the FE model was set to 1500 kg/m^3^, consistent with published laminate data for these systems [39,42].

The orthotropic elastic constants of the pristine and damage-simulated overwrap are summarized in Table 2.

**Figure 5:**
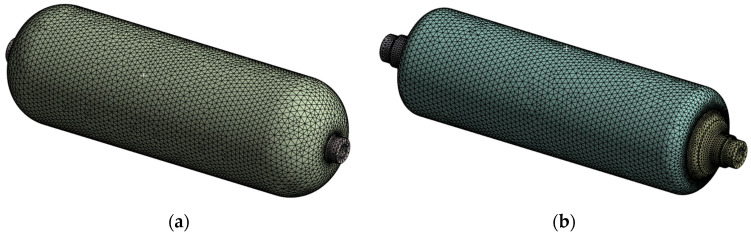
Mesh density and sub-meshes. (**a**) Outer carbon/epoxy shell (88,763 quadratic tetrahedra); (**b**) Inner HDPE liner and aluminum bosses (84,863 elements). Local refinement was introduced around material interfaces and the dent.

Homogenizing the filament-wound overwrap into an equivalent orthotropic continuum preserves the overall layup thickness and mass distribution and, therefore, does not materially alter the elastic eigenfrequencies and mode shapes. The liner was kept undamaged; damage was introduced only in the overwrap. The impact-affected region was idealized as a cylindrical sub-volume in which all orthotropic elastic constants were uniformly scaled by 0.1. This effective-stiffness representation replaces microcracking and short delamination in surface plies with an equivalent weakened layer; the associated bending-response degradation was validated against 3-D finite-element simulations and tests and, at high damage densities, approached the classical ply-discount limit [31]. Adopting a pronounced knockdown as a conservative sensitivity case is consistent with engineering practice used in preliminary analyses when ply-scale morphology is unknown [44]. Classical vibration studies further show that the observability of delamination in natural frequencies, mode shapes, and damping increases with damage severity, whereas small defects can remain globally indistinct [45]. For composite cylindrical shells, a bending-dominated response is particularly sensitive to interlaminar degradation, which supports using a strong reduction to ensure detectability in modal screening before exploring milder reductions [32]. This conservative stiffness knockdown is a common surrogate for BVID in FE modeling and avoids explicit ply-level cracking [25,46]. Because the degraded zone is embedded in the continuum, its influence on global mode shapes arises naturally without auxiliary springs or contact elements. The geometry was discretized with quadratic tetrahedra (10-node). The global element size was set by a mesh-convergence study, and the local element size was reduced near material interfaces and around the damaged sub-volume (Figure 5a,b), yielding 173,626 elements. Subdomains were coupled with bonded contacts to represent co-cured or interference-fit interfaces. Free–free boundary conditions reproduced the support-free configuration of the roving hammer test, and the first 60 natural modes were extracted.

**Figure 6:**
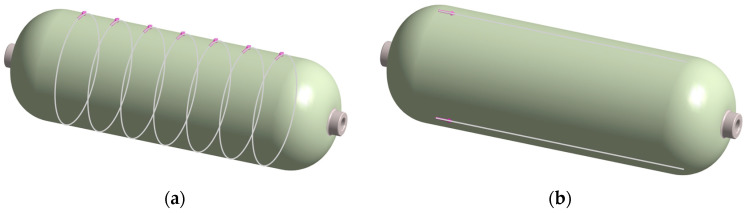
Virtual scan lines for shape analysis. (**a**) Seven circumferential rings, evenly spaced around the vessel. One ring passes through the impact center. (**b**) Longitudinal lines along the vessel axis. Radial displacement was sampled along these paths to form 1D profiles.

To facilitate shape-based diagnostics, virtual scan lines were embedded on the external surface prior to meshing: two longitudinal meridians and seven circumferential rings, with one ring centered on the dent location (Figure 6). For every computed mode, the normal surface displacement was sampled along each path. The resulting one-dimensional profiles provide cross-sections of the global mode shapes; for each mode, pristine and damaged profiles were contrasted along the same scan lines to compare trends and spatial patterns and to visualize the influence of damage.

To verify that mode-shape changes stem solely from impact-induced stiffness loss, the Modal Assurance Criterion (MAC) was evaluated only for matched modal indices of the pristine finite-element (FE) model $\phi_{r}^{0}$ and the impact-damaged model $\phi_{r}^{d}$. After mass-normalizing both eigenvectors, the diagonal MAC for the *r*-th mode reduces to:

(1) $$MAC_{r}=\frac{{\left|\phi_{r}^{0T}\phi_{r}^{d}\right|}^{2}}{{\left\|\phi_{r}^{0}\right\|}^{2}{\left\|\phi_{r}^{d}\right\|}^{2}}$$

Equation (1) yields a scalar between 0 and 1; values close to 1 indicate an unchanged mode shape. The interpretation and uncertainty of MAC are discussed in [26,27].

To establish an independent, shape-based diagnostic and to define a priori expectations for the experiment, the FE model’s mode shapes were evaluated along a predefined network of virtual scan lines on the external surface. For each mode, the normal-to-surface component of the mode shape (eigenvector) was sampled at mesh nodes along each path and reparametrized by the arc-length coordinate s to form a one-dimensional profile p(s); amplitudes follow the arbitrary normalization of eigenvectors (2). Profiles for the pristine and damaged configurations were then contrasted via the pristine–damaged difference Δp(s) and inspected for trend-level amplitude and curvature changes; these profiles constitute the expected spatial signatures to be confronted with the roving hammer measurements. This line-based comparison is consistent with mode-shape difference strategies (MSDI) that reveal small, spatially coherent deviations between pristine and damaged shapes [47]. Curvature-focused diagnostics (e.g., mode-shape-difference curvature) sharpen bending anomalies and scale with damage size, improving localization relative to displacement or MAC alone [48,49]. These derivative-based features are also less confounded by temperature than frequency shifts, increasing robustness under operational variability [50]. Finally, the path-restricted curvature has a direct energetic interpretation via element-relative modal strain energy, which underpins practical damage indices used in recent two-stage identification frameworks [51].

(2) $$∆p\left(s\right)=p^{d}\left(s\right)-p^{0}(s)$$

This approach reveals local amplifications and phase lags that the diagonal MAC alone does not capture. Screening Δp(s) along the predefined paths predicts the spatial fingerprint of the defect. Restricting the comparison to one-dimensional paths mitigates spatial sampling aliasing when a dense numerical mesh is matched to a sparser experimental grid. Moreover, line-integrated observables (modal curvature and path-restricted strain energy density) have repeatedly proven more sensitive to barely visible impact damage in composite shells than global MAC alone [15,25,30]. Nodal values of the normal-to-surface component of the mode-shape vector were exported directly from ANSYS Mechanical and mapped to the arc-length coordinate, so that p(s) represents this component relative to the undeformed cylindrical surface and can be overlaid on experimental polar plots without further transformation. All processing steps were automated in Python (NumPy, Pandas), ensuring reproducible treatment of the full set of sixty modes and enabling rapid, systematic comparison of hundreds of deformation profiles while preserving the spatial resolution required to detect barely visible impact damage.

**Figure 7:**
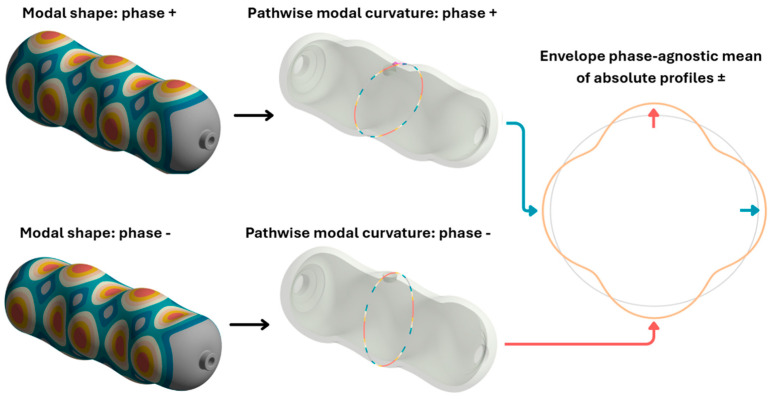
Procedure for obtaining path-line descriptors from FE eigenmodes: phase ± renderings (**left**); sampling the normal-to-surface component of the mode-shape vector along a circumferential scan line to form the signed profile and its path-wise curvature (**middle**); phase-agnostic envelope as the mean of absolute phase ± profiles for FE polar comparison (**right**).

Figure 7 provides a schematic representation of the plotting and assembly workflow.

## 3. Results

The dynamic response of the type-IV composite-overwrapped pressure vessel was characterized using experimental modal analysis. From the averaged frequency response functions (FRFs), sixteen resonances below 5 kHz were identified. The identified natural frequencies are listed in Table 3.

**Figure 8:**
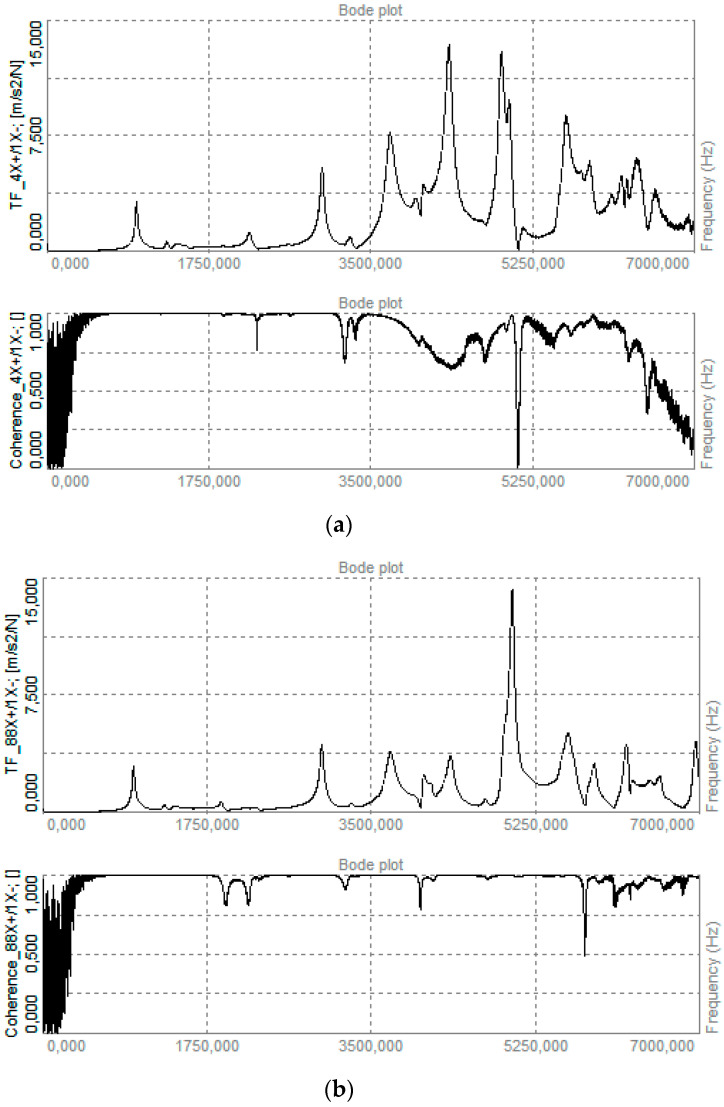
Frequency response characteristics and coherence obtained for the tested vessel: (**a**) Point 4 (impact location), presenting the frequency response function (**top**) and coherence (**bottom**); (**b**) Point 88 (opposite to the impact location), presenting the frequency response function (**top**) and coherence (**bottom**). These plots highlight the differences in dynamic behavior between the impacted and non-impacted regions.

The frequency response functions (FRFs) and coherence spectra recorded at the impact location (Point 4 on Ring 4) and on the diametrically opposite side of the vessel (Point 88 on Ring 4) are presented in Figure 8. In both cases, the coherence remained at a satisfactory level. The FRF magnitude at Point 4 exhibited pronounced peaks at 3695, 4340, and 4901 Hz, whereas the corresponding peaks at Point 88 were of significantly lower amplitude. The remaining resonances showed comparable levels at the two positions.

**Figure 9:**
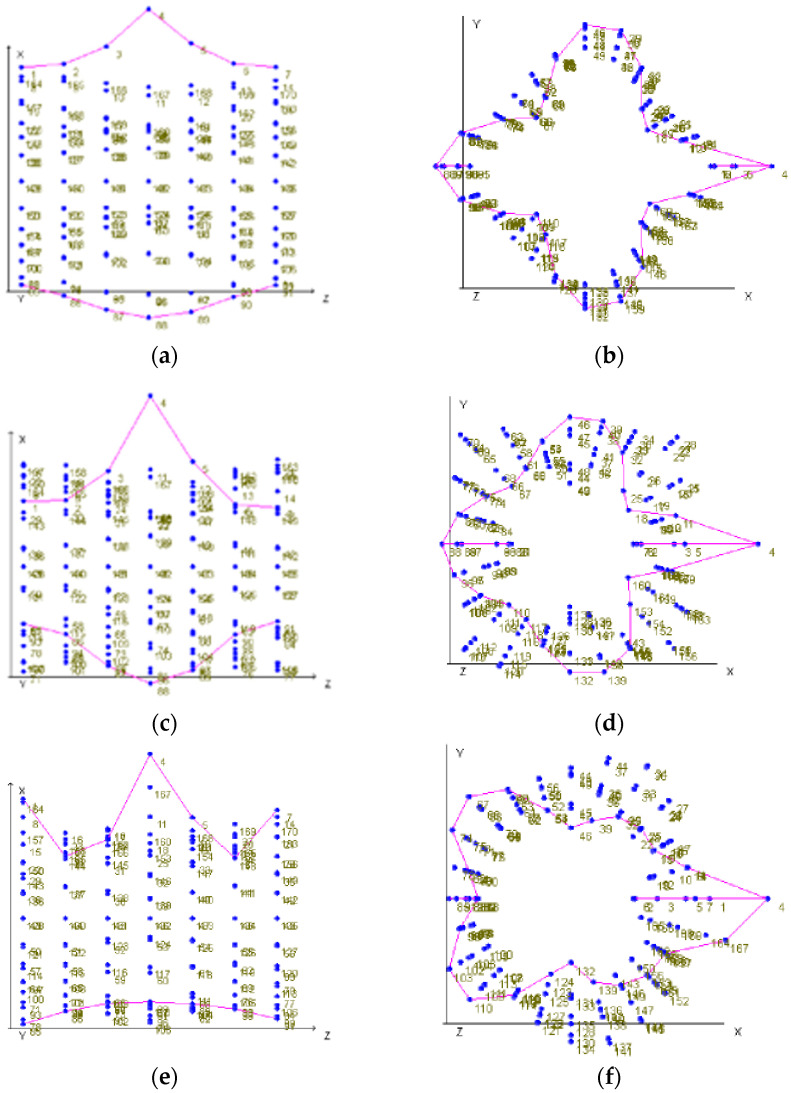
Mode shapes of the vessel obtained at various frequencies and views. (**a**,**b**): Vibration forms at 3695 Hz, showing deformation perpendicular (**a**) and along the vessel axis (**b**). (**c**,**d**): Vibration forms at 4340 Hz, showing deformation perpendicular (**c**) and along the vessel axis (**d**). (**e**,**f**): Vibration forms at 4901 Hz, showing deformation perpendicular (**e**) and along the vessel axis (**f**).

Mode shapes reconstructed from FRFs at 168 excitation points show increasing geometric complexity with frequency. Representative shapes at 3695, 4340, and 4901 Hz are shown in Figure 9. Each figure presents the deformation envelope in two views: left perpendicular to the cylinder axis and right aligned with the axis. For each mode, the pink polyline is the reconstructed radial displacement along Ring 4, and the blue markers indicate the grid points used for roving hammer excitation. The 3695 Hz and 4340 Hz modes form four-lobe patterns with alternating outward and inward motion. The 4901 Hz mode forms a five-lobe pattern. In all cases, the largest radial amplitudes occur along the longitudinal path that crosses the impact meridian, while the opposite meridian remains close to a baseline. This pattern matches the FRF amplitude and modal shape asymmetry between the impacted and non-impacted sides.

The Modal Assurance Criterion (MAC) matrix calculated between the undamaged model and the model with a localized stiffness reduction showed that, for the majority of the 60 examined modes, a high shape correlation was preserved (MAC ≥ 0.90), confirming that the global modal order is maintained after impact. The MAC decreases in the 0.49–0.88 range occur in frequency bands with increased modal density, i.e., where the undamaged configuration featured almost degenerate modal pairs. In these bands the local weakening of the shell induces a rotation of the eigenvector within the modal subspace and a partial relocation of the modal amplitudes toward the impacted sector, which results in a lower MAC value for the pair with the same index. Importantly, in such cases, the MAC drop is driven primarily by the very small frequency separation and by the rotation within a two-dimensional, nearly repeated eigenspace, rather than by an actual loss of physical similarity; the correct correspondence may reside in the adjacent off-diagonal MAC entry, so the full 2 × 2 MAC block must be interpreted instead of a single diagonal scalar. This effect is particularly visible for modes 17–18, 21–22, 35–36, 48–49, and 59. Despite these local reductions, the modes exhibiting the strongest amplitude disturbance in the damaged region still reached MAC > 0.90, which confirms the limited sensitivity of classical, diagonal MAC to highly localized stiffness losses in composite pressure vessels. For this reason, the MAC-based evaluation was complemented with line-based displacement-envelope comparisons and curvature-related indicators, which better capture the spatial signature of BVID. A summary of the frequency shifts and MAC values for all modes is provided in Table 4.

**Figure 10:**
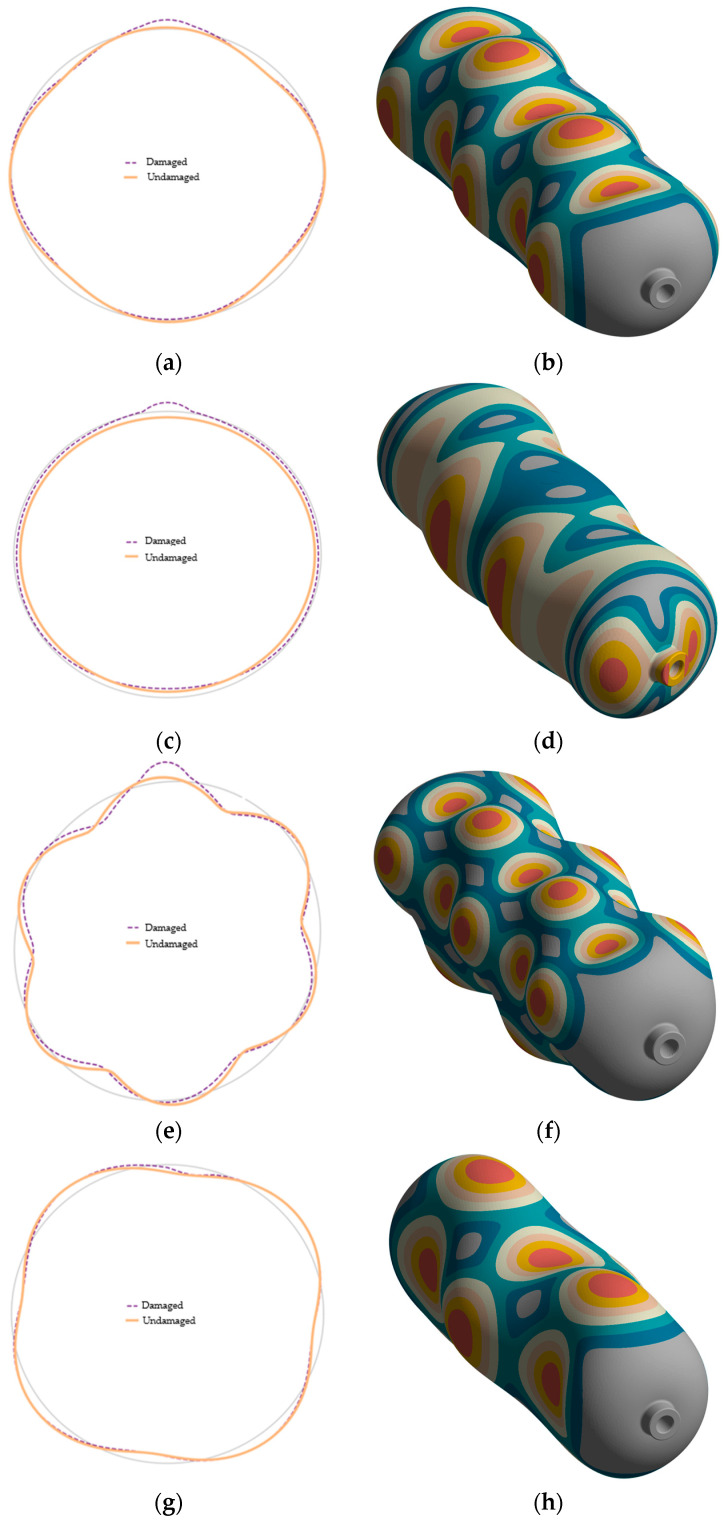
FE radial envelopes for pristine (orange) and damaged (dashed) configurations on the ring passing through the impact meridian, with corresponding mode shapes shown on the right. Panels: (**a**,**b**) mode 23; (**c**,**d**) mode 46; (**e**,**f**) mode 41; (**g**,**h**) mode 12. Local amplitude growth is visible in modes whose antinodes intersect the damaged sector.

Circumferential envelopes of the normal-to-surface component of the mode-shape vector, extracted along the virtual scan lines on the ring passing through the impact meridian (Section 2.2), show that for most modes the undamaged and damaged profiles coincide over the undisturbed part of the circumference, whereas the damaged model exhibits a systematic amplitude increase within the impact sector. This behavior is visible in Figure 10a–h: for the mid-order mode 23 (Figure 10a,b), the deviation is confined to the damaged sector and the circumferential phasing is preserved; for the higher-order circumferential modes 41 and 46 (Figure 10c–f), the same local amplitude growth is accompanied by a slight angular shift of the peak toward the weakened region, indicating a rotation of the modal vector within a nearly degenerate two-mode subspace indicated by the MAC analysis; for the low-order mode 12 (Figure 10g,h), the local effect is superposed on a mild global increase in compliance. The effect is most pronounced for higher-order circumferential modes whose antinodes intersect the damaged zone (e.g., 41 and 46), whereas for mid-order modes (e.g., 23), the perturbation remains localized and does not change the global phasing. These scan-line results therefore expose spatially confined stiffness loss that the diagonal MAC alone does not capture and are fully consistent with the subspace rotation mechanism inferred from the MAC matrix.

## 4. Discussion

Combining frequency response function (FRF) features with eigenvector-based metrics allowed us to localize the barely visible impact damage in the COPV. In the experimental modal analysis, a distinct asymmetry was observed at approximately 3.70, 4.34, and 4.90 kHz; the FRFs measured at the impact meridian (Point 4) showed higher response amplitudes and lower coherence compared to those at the diametrically opposite meridian (Point 88). This pattern of elevated vibrational response at the damaged location matches damage signatures reported in the structural health monitoring literature [5,15]. Moreover, the finite element (FE) model updated with a localized 90% stiffness reduction in the overwrap reproduced the same FRF amplitude and coherence trends, confirming that the observed asymmetry was driven by a local loss of circumferential stiffness rather than by measurement noise.

For the most part, introducing the local stiffness loss did not disrupt the vessel’s modal structure. The modal assurance criterion (MAC) comparison between the intact COPV and the locally “damaged” model showed that about two-thirds of the first 60 modes retained a high correlation (MAC ≥ 0.90), indicating the global mode order was preserved and the damage did not significantly reorganize the spectrum. However, noticeably lower MAC values (ranging from 0.49 to 0.88) were found almost exclusively in five clusters of nearly degenerate circumferential modes (specifically the mode pairs 17–18, 21–22, 35–36, 48–49, and the singlet mode 59). In these cases, the impact did not fundamentally change the deformation pattern but instead rotated the eigenvectors within their shared two-dimensional modal subspace—exactly the limitation of diagonal MAC noted by Allemang and later quantified by Greś, Döhler, and Mevel [26,27]. This phenomenon implies that a simple one-to-one MAC comparison can misidentify mode correspondences when damage induces such eigenvector rotation. Accordingly, to correctly pair the modes in those clusters, one must evaluate the full 2 × 2 MAC matrix for each degenerate pair and select the pairing that yields the highest correlation.

Further insight was provided by examining radial displacement scan-line profiles around the vessel’s circumference, which clearly reflected the presence of the impact. Mode shapes whose antinodal regions intersect the damaged sector (for example, modes 41 and 46) displayed a pronounced local amplitude bulge and a slight angular shift of the peak toward the impact site, whereas the rest of the circumferential profile remained unchanged. A representative mid-order mode (mode 23) was only minimally affected in the immediate vicinity of the dent, and a lower-order mode (mode 12) exhibited a slight overall compliance increase in addition to its localized deformation around the damage. This behavior is consistent with damage metrics based on mode shape curvature or modal strain energy in composite structures, wherein the presence of damage produces localized distortions that may not be evident from a coarse global indicator like diagonal MAC [15,25,30].

In summary, while MAC can serve as a rapid global screening and mode-pairing tool in this application, it must be complemented by higher-resolution spatial damage descriptors (such as mode shape scan-lines or curvature/MSE maps) and, when available, by FRF asymmetry measures. This combined multi-metric approach aligns with recommendations from other vibration-based damage detection studies on composite and filament-wound shells [4,9,13,29,30].

## 5. Conclusions

This study demonstrates that a combined experimental–numerical modal analysis approach can detect and circumferentially localize barely visible impact damage in a type-IV composite pressure vessel. In our tests, hammer-impact excitation over a 168-point grid revealed a repeatable pattern of increased FRF amplitude and reduced coherence at the impact site, and an FE model incorporating a local 90% stiffness knockdown reproduced the same circumferential response asymmetry. This agreement confirms that the observed FRF anomalies were caused by a genuine local stiffness loss in the structure rather than by measurement noise.

Modal correlation analysis indicated that the damage had minimal effect on most mode shapes; for a majority of the 60 analyzed modes, the diagonal MAC between the pristine and damaged models remained above 0.90. However, the MAC dropped into the 0.49–0.88 range for several nearly degenerate circumferential mode pairs, reflecting that the impact did not alter these modes’ overall deformation mechanism but instead rotated their mode vectors within a common subspace [26,27]. Consequently, while MAC was effective for global mode matching and initial screening, it could not serve as a standalone damage detector in this scenario.

Detailed examination of the mode shapes provided the spatial resolution that the MAC metric lacked. Notably, modes with antinodes passing through the damaged sector showed a clear localized bulge in their displacement envelope and a slight angular shift of the peak toward the impact point, consistent with curvature- and strain energy-based damage indicators for composites [13,15,25,30]. Based on these observations, a practical damage-identification workflow emerges: FRF amplitude/coherence asymmetry serves as a global damage indicator; MAC analysis is used for mode clustering and pairing; and mode shape scan-lines or curvature/MSE maps provide the final high-resolution localization of the damage.

As a note of caution, the lowest circumferential mode predicted by the FE model (around 0.6 kHz) was not observed in the experiment, so the test–simulation comparison could only be made for higher-frequency modes and in terms of overall trends rather than one-to-one mode matching. This limitation should be clearly acknowledged when applying the method in practice. Moreover, the present results are based on a single impacted vessel, using a homogenized overwrap model with an assumed damage extent and an imposed stiffness reduction. The proposed procedure should therefore be further validated on composite pressure vessels with different layups, impact energies, and damage extents, ideally informed by non-destructive inspection data to calibrate the damage parameters.

## Figures and Tables

**Table 1:** Physical properties of the materials used.

Material	Density $\rho \;[kg/m^{3}]$	Young Modulus E [GPa]	Poisson Ratio v [−]
Aluminum (bosses)	2700	70.0	0.33
HDPE (liner)	840	1.2	0.40

**Table 2:** Orthotropic elastic constants for the carbon/epoxy overwrap used in the modal FE model. The hoop direction (θ) corresponds to the filament-wound circumferential direction; axial (z) is along the vessel; radial (r) is through the thickness.

Material	ρ $\left[kg/m^{3}\right]$	$E_{z}$ [GPa]	$E_{\theta }$ [GPa]	$E_{r}$ [GPa]	$v_{\theta r}$ [−]	$v_{\theta z}$ [−]	$v_{zr}$ [−]	$G_{\theta z}$ [GPa]	$G_{\theta r}$ [GPa]	$G_{zr}$ [GPa]
Homogenized Carbon Overwrap (pristine)	1500	8.0	140.0	8.0	0.02	0.34	0.02	5.0	5.0	5.0
Homogenized Carbon Overwrap (damaged)	1500	0.8	14.0	0.8	0.02	0.34	0.02	0.5	0.5	0.5

**Table 3:** Experimentally identified natural frequencies.

Mode Number	Frequency [Hz]
1	965
2	1278
3	1371
4	1508
5	1899
6	2184
7	2623
8	2969
9	3215
10	3695
11	3973
12	4050
13	4145
14	4340
15	4708
16	4901

**Table 4:** Comparison of modal frequencies for the pristine and damaged configurations with the diagonal Modal Assurance Criterion (MAC).

Mode	Undamaged Model [Hz]	Damaged Model [Hz]	∆f	MAC	Mode	Undamaged Model [Hz]	Damaged Model [Hz]	∆f	MAC
1	588.80	584.30	4.51	1.00	31	2811.14	2810.85	0.28	0.84
2	588.78	588.76	0.02	1.00	32	2848.10	2839.02	9.08	0.95
3	964.13	959.23	4.90	1.00	33	2920.41	2901.78	18.63	0.93
4	969.92	966.69	3.22	1.00	34	2920.43	2904.74	15.69	0.93
5	1123.69	1112.90	10.79	0.88	35	2993.57	2991.71	1.86	0.64
6	1123.71	1119.71	4.00	0.88	36	2993.43	2992.97	0.46	0.65
7	1260.71	1250.01	10.70	0.98	37	3118.36	3115.07	3.30	0.98
8	1260.67	1260.56	0.11	1.00	38	3118.23	3116.90	1.33	1.00
9	1273.43	1272.76	0.67	0.98	39	3170.94	3154.56	16.39	0.97
10	1273.37	1273.22	0.16	1.00	40	3170.68	3168.64	2.04	1.00
11	1616.20	1604.77	11.44	0.99	41	3402.69	3381.20	21.49	0.96
12	1616.24	1613.11	3.13	0.99	42	3402.79	3386.50	16.29	0.96
13	1874.77	1874.69	0.08	1.00	43	3606.34	3597.23	9.12	0.97
14	1916.72	1916.66	0.06	1.00	44	3606.15	3604.59	1.56	0.98
15	1936.46	1935.05	1.41	0.98	45	3617.90	3617.26	0.64	0.99
16	1936.40	1936.21	0.19	0.98	46	3635.11	3618.79	16.32	0.97
17	1951.59	1949.97	1.62	0.77	47	3634.88	3634.74	0.14	1.00
18	1951.53	1951.43	0.10	0.77	48	3730.18	3717.54	12.64	0.61
19	2085.26	2076.19	9.07	1.00	49	3730.21	3725.98	4.23	0.62
20	2085.08	2084.27	0.81	1.00	50	3753.17	3752.75	0.42	0.83
21	2381.64	2373.65	7.99	0.68	51	3761.85	3752.84	9.01	0.82
22	2381.77	2381.63	0.14	0.68	52	3761.64	3758.28	3.36	1.00
23	2614.70	2601.01	13.69	0.98	53	3872.17	3856.74	15.43	0.98
24	2614.78	2611.20	3.58	0.99	54	3871.98	3870.62	1.37	1.00
25	2728.55	2720.45	8.10	0.99	55	4145.48	4136.28	9.20	0.88
26	2770.36	2724.84	45.52	0.86	56	4145.88	4145.48	0.40	0.99
27	2772.24	2748.48	23.76	0.81	57	4180.98	4159.86	21.12	0.68
28	2772.33	2751.77	20.56	0.82	58	4181.07	4163.01	18.06	0.76
29	2770.28	2769.87	0.41	0.91	59	4284.00	4235.61	48.39	0.49
30	2811.18	2810.74	0.44	0.84	60	4283.88	4282.43	1.46	0.96

## Data Availability

The raw data supporting the conclusions of this article will be made available by the authors on request.
